# Silymarin and Silybin: Rejuvenating Traditional Remedies with Modern Delivery Strategies

**DOI:** 10.3390/pharmaceutics17121628

**Published:** 2025-12-18

**Authors:** Xiuyan Li, Han Zhu, Yanhong Wang, Xiwu Zhang, Zhixin Yang, Xueying Yan, Qin Yu

**Affiliations:** 1 Key Laboratory of Basic and Application Research of Beiyao, Heilongjiang University of Chinese Medicine, Harbin 150001, China; lixiuyan211486@163.com (X.L.); wang.yanhong@163.com (Y.W.); zhixin.y@163.com (Z.Y.); 2 Department of Pharmacy, Shanghai Pudong Hospital, Fudan University Pudong Medical Center, 2800 Gongwei Road, Pudong, Shanghai 201399, China; han_zhu001@163.com; 3 Experimental Teaching and Practical Training Center, Heilongjiang University of Chinese Medicine, Harbin 150001, China; zxw506zxw@163.com; 4 Shanghai Skin Disease Hospital, Tongji University School of Medicine, Shanghai 200443, China

**Keywords:** silymarin, silybin, milk thistles, bioavailability, biopharmaceutics, nanotechnology

## Abstract

Silymarin, a polyphenolic flavonolignan complex extracted from *Silybum marianum* (milk thistle), has long been recognized for its hepatoprotective, antioxidant, anti-inflammatory, and anticancer properties. Among its constituents, silybin is the most pharmacologically active and has been extensively studied in both preclinical and clinical settings. However, the clinical application of silymarin-based therapies remains limited by poor aqueous solubility, low oral bioavailability, rapid metabolism, and physicochemical instability. This review systematically outlines the pharmacokinetic challenges of silymarin and highlights recent advancements in formulation strategies designed to overcome these barriers. Key innovations include nanotechnology-enabled delivery systems, lipid-based carriers, water-soluble derivatives, bioavailability enhancers, parenteral and transdermal formulations, as well as controlled and synchronous release technologies. These approaches significantly improve tissue targeting, intracellular uptake, and pharmacological efficacy. Additionally, this review evaluates currently marketed silymarin formulations and recent clinical/preclinical evidence, revealing a persistent gap between laboratory advances and commercially available products. By synthesizing the mechanistic, regulatory, and manufacturability barriers that hinder translation, we delineate the key challenges that must be addressed to enable clinically deployable next-generation silymarin products. Collectively, these insights illustrate a paradigm shift in the modernization of phytomedicine, positioning silymarin as a model compound for the transformation of traditional herbal remedies into precision therapeutics through interdisciplinary drug delivery innovations.

## 1. Introduction

**Figure 1:**
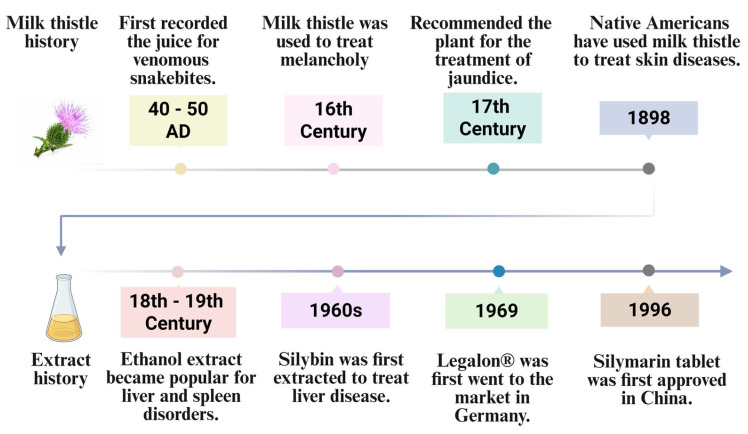
Historical and clinical evolution of milk thistles. This figure illustrates two parallel timelines: the historical use of the milk thistle, and the development of its modern pharmaceutical preparations. (Up) Historical Use of Milk Thistle: Documented applications range from the use of its juice to treat snakebites (1st century AD) to its employment by Native Americans for skin diseases (late 19th century). (Down) Development of Extracts: The evolution spans from popular ethanol extracts for liver and spleen disorders (18th–19th century) to the first regulatory approval of a silymarin tablet (China, 1996) (Created in BioRender. Li, X. (2025) https://BioRender.com/6jgwx79).

Traditional herbal medicines have played a vital role in healthcare for centuries, offering diverse therapeutic benefits across cultures. *Silybum marianum* (milk thistle), a flowering plant native to the Mediterranean, is particularly renowned for its longstanding use in liver-related disorders [1,2,3,4]. The genus name Silybum was designated by the ancient Greek physician Dioscorides, while the species name marianum derives from Christian legend, in which drops of the Virgin Mary’s milk fell upon thistle leaves, leaving distinctive white markings [1,5,6]. Owing to its high adaptability, milk thistle has since spread across Europe, the America, South Australia, India, and China, and is even classified as an invasive species in some regions [1]. Historical records trace the medicinal use of milk thistle back over 2000 years. Dioscorides first documented it as a treat for venomous snakebites around 40–50 A.D. [1,7]. During the 16th century, British herbalists prescribed it for melancholy [5], and in the 17th century, Nicholas Culpeper recommended it for jaundice and hepatic obstructions [3]. In 19th-century North America, Eclectic physicians used it for liver, spleen, and kidney congestion, while Native American traditions extended its application to skin diseases and boils [7]. Folk remedies utilized various parts of the plant (e.g., leaves, flowers, and seeds) to treat ailments ranging from gallstones and hemorrhages to bronchitis and peritonitis. “Rademacher’s tincture,” a popular 18th- and 19th-century ethanolic extract of milk thistle, was widely used for liver and spleen disorders [3]. Scientific interest surged in the 20th century, particularly in Germany, leading to formal approval of milk thistle-based formulations by the German Commission E in the 1960s [3]. Since then, silymarin-containing products have achieved widespread global recognition as both pharmaceuticals and dietary supplements [1,2,8,9,10,11,12,13,14] (Figure 1).

**Figure 2:**
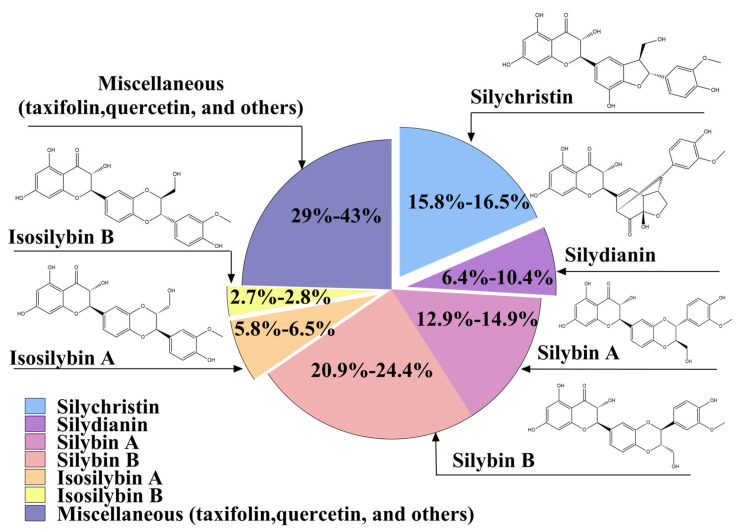
The ratio of components in silymarin extraction. This pie chart quantifies the typical percentage distribution of the seven primary flavonolignan groups in silymarin. The silybin isomers (A and B) constitute the most abundant fraction (33–39%), followed by isosilybin A/B, silychristin, and silydianin. Other minor or unspecified flavonolignans collectively account for 29–43% of the total extract.

The primary bioactive extract of milk thistle is silymarin, a complex mixture of flavonolignans derived from the plant’s seeds and accounting for over 60% of the extract’s dry weight. Its major constituents include silybin (also known as silibinin), silychristin, silydianin, and isosilybin, with silybin comprising approximately 30% and exerting the strongest pharmacological activity. Structurally, silybin features rigid polycyclic frameworks with heterocyclic moieties, rendering it chemically stable under acidic conditions but prone to oxidation (e.g., forming 2,3-dehydrosilybin) or degradation in alkaline or Lewis acid environments [2]. Silychristin and silydianin contribute approximately 20% and 10%, respectively, to the total silymarin content [15,16] (Figure 2). 

Modern pharmacological studies have confirmed the broad-spectrum bioactivity of silymarin and silybin, particularly their hepatoprotective properties [17,18,19]. These effects are mediated through inhibition of lipid peroxidation, free radical scavenging, and upregulation of endogenous antioxidant enzymes. Clinically, silymarin has shown promise in treating alcoholic and non-alcoholic fatty liver disease (NAFLD) [20,21], hepatitis-induced liver injury [22], and drug-induced hepatotoxicity. Beyond hepatology, its therapeutic potential extends to neurotoxicity [23], atherosclerosis [24], chronic obstructive pulmonary disease (COPD) [24], renal and cardiovascular diseases [25,26], Alzheimer’s disease [27], and other neurodegenerative conditions [28]. Furthermore, anticancer activity has been reported in preclinical studies on pancreatic, gastric, and colorectal malignancies [29].

Despite these promising bioactivities and excellent clinical safety profiles (with daily doses up to 1500 mg being well tolerated) [30,31], the therapeutic translation of silymarin and silybin remains limited due to several pharmaceutical barriers. These include poor aqueous solubility (50–430 μg/mL), low membrane permeability, extensive first-pass metabolism, and instability in physiological and formulation conditions [32,33,34,35]. For instance, the absolute oral bioavailability of silybin is as low as 0.45%, and even its maximal solubility in polyethylene glycol 400 (PEG 400) (0.243 g/mL) is insufficient for effective liquid dosage forms [36,37]. Though isolated silybin (e.g., silybin meglumine) can enhance water solubility, it often accelerates metabolism and shortens the therapeutic window. Moreover, separation from the whole silymarin matrix may disrupt synergistic effects between components, potentially compromising efficacy, absorption, and stability [32,38].

To overcome these challenges, a wide array of modern drug delivery strategies, such as nanocarrier-based systems, lipid-based vehicles, and targeted delivery platforms, have been developed to improve the solubility, absorption, biodistribution, and overall therapeutic performance of silymarin. Several review articles have previously summarized advances in silymarin delivery; however, most focus on a single class of carrier, lack a comparative biopharmaceutical analysis, or do not integrate developments across oral, parenteral, and targeted delivery platforms [1]. To provide an updated and quantitatively grounded perspective, we systematically surveyed the literature published from 1998 to 2025, screening more than 300 original research articles, patents, and clinical reports related to silymarin and silybin delivery systems. Among these, over 170 studies involved nanoparticle-based formulations, including lipid nanoparticles, nanoemulsions, polymeric nanocarriers, cyclodextrin-associated hybrid systems, and other emerging nanostructured platforms. This quantitative mapping highlights the rapid expansion of nanotechnology-enabled approaches over the past two decades and underscores the need to critically distinguish which strategies offer genuine biopharmaceutical advantages versus incremental formulation modifications. By integrating phytochemical characteristics, nanotechnological advances, and biopharmaceutics principles, this review aims to delineate the current landscape of silymarin delivery research, identify the mechanistic basis underlying successful systems, and clarify the remaining translational barriers. Collectively, this updated analysis establishes a framework for guiding future formulation design toward clinically meaningful improvements in the therapeutic performance of silymarin.

## 2. Advancements in Research on Rejuvenating Traditional Remedies

Silymarin, comprising silybin and a series of polyphenolic compounds, has long been recognized for its potent antioxidant and hepatoprotective properties [8,12,39]. Over the past few decades, extensive preclinical and clinical investigations have expanded its therapeutic profile far beyond traditional hepatobiliary indications. Mechanistic studies have uncovered its roles in free radical scavenging, membrane stabilization, glutathione modulation, inhibition of inflammatory pathways, antifibrotic effect, regulation of cell proliferation, and modulation of apoptotic properties [8,17,39,40,41,42,43,44,45]. These mechanistic insights have expanded interest in its use beyond traditional hepatobiliary disorders.

Extensive preclinical and clinical investigations now demonstrate silymarin’s benefits in acute and chronic hepatic injury [24], mitigation of chemotherapy-related organ toxicity [46,47], and protection of cardiovascular and neurological function [25,27,48]. Additionally, silymarin exhibits hypoglycemic and lipid-lowering effects, contributing to therapeutic potential in diabetes, dyslipidemia, and metabolic syndrome [49,50,51]. Its utility as an adjuvant to anticancer therapy, particularly in reducing hepatotoxicity, gastrointestinal disturbances, and treatment-related discomfort, has further expanded its clinical interest [52,53,54]. Increasing evidence also suggests potential benefits in neurodegenerative diseases, malignancies, metabolic dysfunction, and inflammatory disorders [13,14,45,55,56,57,58].

Despite this expanding broadening therapeutic landscape, an analysis of the clinical research pipeline reveals a significant gap between therapeutic exploration and formulation innovation. According to the latest Medidata registry, more than 60 clinical studies evaluating silymarin or silybin were conducted from 2005 to 2025. Approximately half focus on liver-related diseases, including hepatitis C, acute viral hepatitis (A/B/C), NAFLD, and drug-induced liver injury, while others address immune dysfunction (e.g., HIV infection, immune abnormalities), infectious diseases such as COVID-19 pneumonia, and dermatologic conditions like acne vulgaris and melasma. A smaller portion investigates applications in cancer or severe systemic diseases requiring intravenous administration.

However, the overwhelming majority of these trials rely on conventional oral standardized silymarin extracts, with only sporadic exploration of topical dermatologic formulations. Remarkably, only one known study has evaluated a next-generation delivery system: the micellar formulation LipoMicel^®^ (NCT06882681, initiated in 2024), a bioavailability study whose results are still pending. High-end pharmaceutical technologies, such as nano-delivery systems, solid dispersions, improved salt forms, and phospholipid-based complexes, remain largely absent from the clinical trial landscape, despite strong preclinical evidence demonstrating substantial enhancements in solubility, absorption, and systemic exposure. Table 1 summarizes key recent clinical studies and highlights the types of formulations, dosage regimens, and primary outcomes currently under evaluation.

This discrepancy highlights a persistent translational bottleneck. Although multiple reviews have discussed isolated formulation strategies [30,34,64,65,66,67], few provide a comparative, biopharmaceutically oriented synthesis that integrates oral, parenteral, and nanotechnology-enabled delivery systems. Consequently, there is a clear need for an updated, mechanism-driven assessment of modern formulation approaches that can support the development of clinically meaningful, pharmacokinetically optimized silymarin products.

## 3. Motivations for Developing New Formulation Strategies

### 3.1. Biopharmaceutical Barriers Restricting the Pharmacological Activity of Silymarin

**Figure 3:**
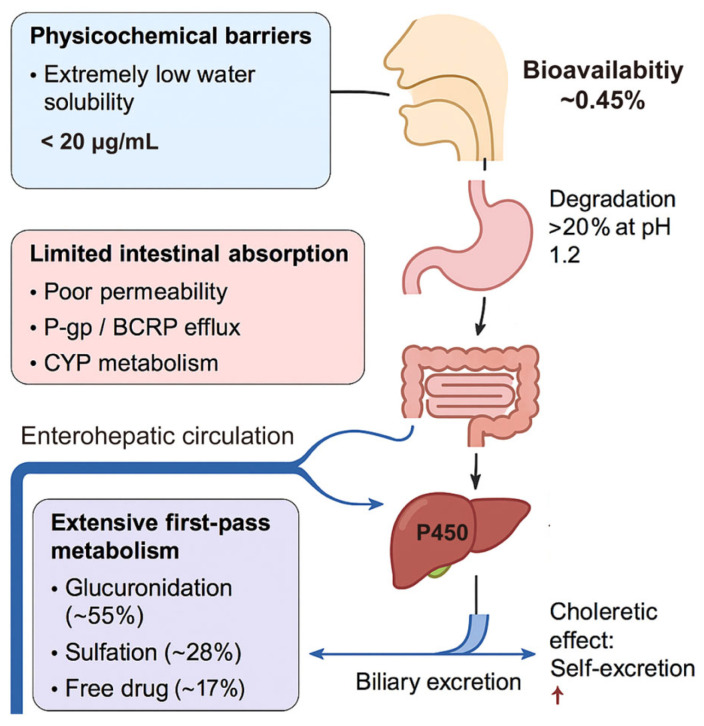
Biopharmaceutical barriers limiting the oral bioavailability of silymarin. Oral absorption of silymarin, particularly silybin, is severely restricted by multiple factors: poor aqueous solubility, low membrane permeability, active efflux by intestinal transporters (e.g., P-gp, BCRP), chemical instability under gastrointestinal pH, extensive first-pass glucuronidation/sulfation, rapid biliary excretion, and pharmacokinetic divergence among flavonolignans. Together, these barriers result in extremely low bioavailability (~0.45%), underscoring the need for formulation strategies that enhance solubility, permeability, efflux inhibition, and metabolic stability. Note: P-glycoprotein (P-gp); breast cancer resistance protein (BCRP); cytochrome P450 (CYP), and ↑ means increased.

Despite its multifaceted pharmacological profile, the clinical effectiveness of silymarin (particularly its key active component, silybin) is significantly hindered by poor biopharmaceutical properties. The absolute oral bioavailability of silybin in humans is strikingly low (~0.45 ± 0.28%), attributable to multiple interrelated factors, including poor aqueous solubility, limited membrane permeability, active intestinal efflux, extensive first-pass hepatic metabolism, and accelerated biliary excretion (Figure 3) [33].

At the gastrointestinal level, silybin faces three major obstacles. First, its extremely low aqueous solubility (<20 μg/mL at 35 °C) leads to incomplete dissolution in gastrointestinal fluids [68]. Second, intestinal permeability studies have yielded intermediate values (apparent permeability coefficient (Papp) 10^−6^–10^−5^ cm/s), falling short of the threshold for high permeability [69]. Third, in addition to poor passive diffusion, silybin is actively transported out of enterocytes by ATP-binding cassette (ABC) efflux pumps, notably P-glycoprotein (P-gp) and breast cancer resistance protein (BCRP). This efflux significantly reduces intestinal absorption, explaining the discrepancy between improved formulations and persistently low systemic exposure. Accordingly, the classification of silybin within the Biopharmaceutics Classification System (BCS) remains debated: while some place it in Class II (low solubility, high permeability) [70], increasing evidence supports Class IV (low solubility, low permeability), given its poor lipophilicity, high efflux liability, and inefficient absorption [34].

Beyond absorption, chemical and metabolic instability further restrict systemic availability. Under simulated gastric conditions (pH 1.2), more than 20% of silybin degrades within two hours [71,72]. Once absorbed, it undergoes rapid phase II conjugation (glucuronidation and sulfation) in both enterocytes and hepatocytes. Plasma pharmacokinetic profiles indicate that conjugated metabolites dominate circulation (~55% glucuronides, ~28% sulfates), while free flavonolignans account for less than 20%. Although conjugates display modestly prolonged half-lives (3–6 h) compared to free silybin (1–3 h), overall systemic retention remains unsatisfactory [16,32].

Silymarin also exhibits unfavorable hepatobiliary handling. Owing to its intrinsic choleretic effect, it stimulates bile flow and thereby accelerates its own excretion. This feedback mechanism, coupled with presystemic metabolism, severely limits oral bioavailability. Attempts to compensate by high dosing frequently result in gastrointestinal adverse effects, such as diarrhea [73]. An additional complication arises from pharmacokinetic divergence among flavonolignans. For instance, silybin B is metabolized more rapidly than silybin A, and other components such as silychristin and silydianin are often undetectable in plasma. This asynchrony hampers dose standardization and may underlie the variable clinical outcomes reported across trials.

Together, these biopharmaceutical hurdles necessitate formulation approaches that synergize solubility enhancement, permeability improvement, efflux inhibition, metabolic stabilization, and optimized tissue distribution. Traditional dosage forms and existing commercial products partially address these issues but remain insufficient to achieve reliable therapeutic exposure, prompting continued exploration of advanced delivery technologies.

### 3.2. Commercially Available Products and Their Limitations

Several silymarin formulations are already marketed worldwide, with Legalon^®^ representing the most widely recognized pharmaceutical-grade product. Common commercial dosage forms include tablets, capsules and dripping pills, which differ substantially in manufacturing techniques, excipient composition, and dissolution performance. Tablets comprise conventional compressed tablets (e.g., silymarin tablets) and dispersible tablets (e.g., Yiganling dispersible tablets). Capsules include soft gelatin capsules (e.g., silymarin soft capsules) and hard-shell capsules (e.g., silymarin capsules). And dripping pills are typically manufactured based on solid dispersion processes to enhance dissolution. Table 2 summarizes the key features, limitations, and bioavailability impact of commercially silymarin products.

Overall, although these products broaden clinical accessibility, most rely on traditional extract-based or minimally modified formulations that do not adequately overcome silymarin’s inherent biopharmaceutical limitations, mainly extremely low aqueous solubility, poor intestinal permeability, and extensive first-pass metabolism. As a result, the oral bioavailability of conventional products remains low, and inter-individual variability in clinical response is common [62,69]. Attempts to improve performance, such as the use of dispersible tablets, solid-dispersion dripping pills, silybin meglumine salts, or phytosome complexes, offer incremental benefits but still fall short of achieving consistent, high systemic exposure [74,75,76,77,78]. Stability issues (e.g., recrystallization in dripping pills), rapid metabolism (e.g., oral salt forms), or higher manufacturing costs (e.g., phospholipid complexes) further limit their broader clinical adoption [76,79,80].

Thus, despite the availability of multiple commercial options, current products provide only modest pharmacokinetic enhancement and have not fully addressed the challenges associated with silymarin delivery. This gap underscores the necessity of developing next-generation formulation strategies, including nanosystems, solid dispersions, micellar platforms, and multifunctional carriers, to achieve reliable therapeutic exposure and unlock silymarin’s full clinical potential.

**Table 2:** Commercial formulation types of silymarin/silybin and their representative products.

Formulation Type	Representative Product(s)	Key Features	Limitations	Reference(s)
Conventional herbal extract	Yi Ganling (China); Legalon^®^ (Germany); Eurosil 85^®^ (Germany);	Silymarin extract; widely used	Poor solubility; variable composition; limited permeability; low bioavailability	[62,69,74,75,76]
Purified silybin formulations	Shui Linjia (China); Natrol Milk Thistle (USA)	Higher silybin content; simple manufacturing	Low solubility; limited absorption	[79]
Salt forms (enhanced solubility)	Silybin meglumine tablets (China); Silybin succinate injection (Legalon^®^ SIL, Germany)	Improved aqueous solubility; IV, bypasses oral absorption barriers	Oral: pH-sensitive; IV: stability, handling issues	[30,77,78]
Phytosome (phospholipid complex)	Siliphos^®^/Silipide^®^(Italy)	Lipid complex enhances permeability	Costly; variable complexation Significant; 4–10× increase vs. standard extract	[30,69,76,81]
Multicomponent nutraceutical blends	Swisse liver detox (Australia); Natrol blends (USA)	Combined botanicals	Contribution of silymarin unclear due to botanical mixtures Uncertain	—

## 4. Advanced Oral Delivery Systems

**Figure 4:**
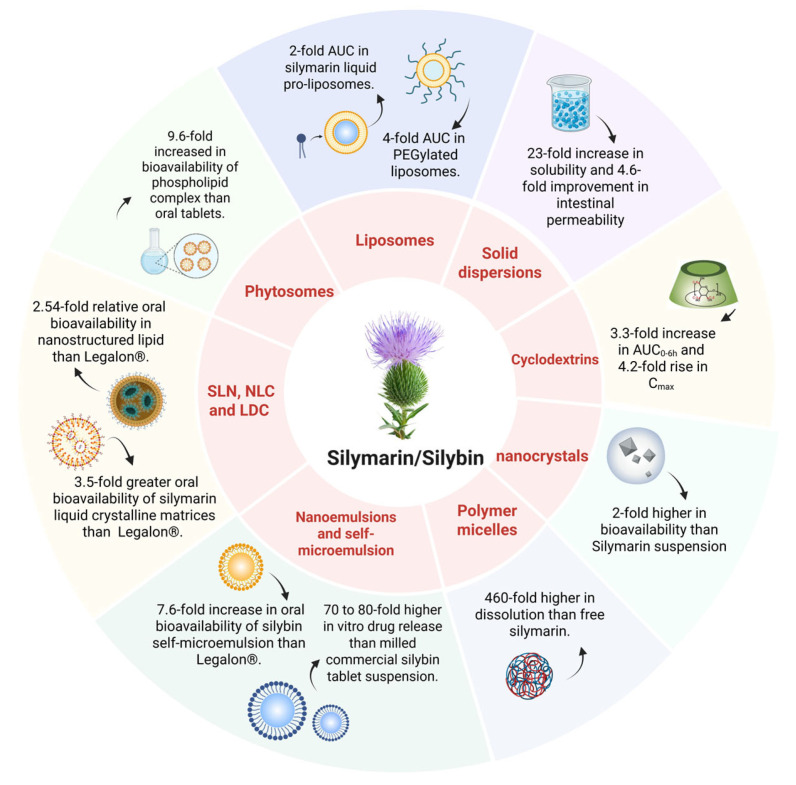
Schematic illustration of advanced oral delivery systems of silymarin, including solid dispersions, cyclodetrins, nanocrystals, lipid-based systems (e.g., liposomes, phytosomes, SLN, NLC, SNEDDS) and polymer micelles. Created in BioRender. Li, X. (2025) https://BioRender.com/f60h278. Note: solid lipid nanoparticle (SLN); nanostructured lipid carrier (NLC); lipid-drug conjugate (LDC).

Despite the availability of multiple commercial formulations, silymarin’s oral bioavailability remains limited due to intrinsic biopharmaceutical barriers, including poor aqueous solubility, low intestinal permeability, active efflux, and extensive first-pass metabolism. To overcome these limitations, a variety of advanced oral delivery systems have been proposed to enhance the absorption and therapeutic efficacy of silymarin. These approaches aim to improve aqueous solubility, increase intestinal permeability, reduce efflux, and mitigate extensive first-pass metabolism. Representative strategies, including solid dispersions, cyclodextrin complexes, particle size reduction, and lipid-based carriers, are summarized in Figure 4 and will be discussed in detail in the following subsections.

### 4.1. Solid Dispersions

Solid dispersion is a widely adopted supersaturating drug delivery strategy aimed at enhancing the solubility and dissolution rate of poorly water-soluble compounds such as silymarin. By dispersing the drug in hydrophilic polymer matrices, this technique generates an amorphous or partially amorphous system that disrupts crystalline packing and inhibits drug recrystallization, thereby facilitating rapid and sustained release in the gastrointestinal environment [82,83,84].

The dissolution behavior of solid dispersions often follows the classic “spring–parachute” profile, where an initial rapid rise in drug concentration (“spring”) is followed by a maintained supersaturation plateau (“parachute”), significantly enhancing absorption potential [83]. Polymers such as hydroxypropyl methylcellulose (HPMC), polyvinylpyrrolidone (PVP), and HPMC acetate succinate (HPMC-AS) are commonly employed as carriers, often combined with surfactants or solubilizing agents (e.g., cyclodextrins) to further stabilize the supersaturated state [85].

In a study by Wei Wu group, a silymarin-PVP solid dispersion was developed using a one-step fluidized bed coating process, in which an ethanolic solution of silymarin and PVP was sprayed onto inert pellets under controlled airflow, yielding uniform coprecipitated layers. This formulation resulted in a 5-fold increase in oral bioavailability in beagle dogs compared to the conventional formulation [80,86]. Similarly, an optimized solid dispersion was prepared via freeze-drying, comprising of silymarin, Tween 80, and hydroxypropyl cellulose (HPC) in a 1:1:3 (*w*/*w*) ratio [87]. This formulation enhanced the relative bioavailability, reaching levels 215% and 589% higher than those achieved with a silybin-excipient mixture or plain silymarin, respectively. Separately, a solid dispersion of silymarin with D-α-tocopherol polyethylene glycol 1000 succinate (TPGS) was developed. It achieved a 3.2-fold improvement in aqueous solubility, along with a significant reduction in both intestinal efflux and glucuronidation, thereby enhancing systemic exposure. Pharmacokinetic evaluations revealed a 4.6-fold improvement in intestinal permeability, accompanied by a marked reduction in efflux ratio (from 5.5 to 0.6) [35].

These biopharmaceutical improvements translated into superior pharmacodynamic outcomes. In vivo studies showed enhanced hepatoprotection in an acetaminophen-induced liver injury model, while in vitro assays demonstrated significantly improved cytotoxicity against lung cancer cell lines, underscoring the formulation’s potential in both hepatic and oncologic indications [88].

### 4.2. Cyclodextrin-Based Inclusion Systems

Cyclodextrins (CDs) are cyclic oligosaccharides composed of α-(6 glucose units), β-(7 units), or γ-(8 units) configurations, characterized by a hydrophobic central cavity and a hydrophilic outer surface. This unique architecture enables the formation of non-covalent inclusion complexes with lipophilic molecules, improving aqueous solubility, chemical stability, mucosal tolerability, and ultimately, systemic bioavailability of poorly water-soluble drugs such as silymarin [89].

Among CD derivatives, β-cyclodextrin (β-CD) and hydroxypropyl-β-cyclodextrin (HP-β-CD) are most frequently utilized in silymarin formulations due to their excellent safety profiles, aqueous solubility, and strong inclusion capacity [90]. Simple CD inclusion complexes have demonstrated clear pharmacological advantages. For instance, β-CD-silymarin complexes exhibited markedly reduced half maximal inhibitory concentration (IC_50_) values in breast cancer cell assays compared with free silymarin, suggesting enhanced cellular uptake and improved therapeutic activity [89]. Furthermore, a sulfhydryl-modified β-CD-silymarin complex achieved substantial improvements in oral pharmacokinetics, showing a 10-fold increase in C_max_ and 9.06-fold elevation in AUC relative to free silymarin, and approximately threefold higher exposure compared with unmodified β-CD complexes. The modified complex attained a relative bioavailability of 39.15%, whereas free silymarin achieved only 4.32%, highlighting the contribution of enhanced mucoadhesion and permeation [91].

Beyond simple inclusion complexes, CDs are frequently integrated into composite nanoparticle systems, which combine the solubilizing effect of CDs with the structural stability, protection, and controlled-release characteristics of nano-carriers. Nanoparticles prepared with silymarin, HPMC, and HP-β-CD produced a 3.3-fold increase in AUC_0–6h_ and 4.2-fold rise in C_max_ compared with unformulated silymarin, indicating substantially enhanced absorption [77]. Similarly, zein-based protein nanoparticles stabilized with β-CD improved silymarin dissolution and oral bioavailability while affording protective encapsulation and sustained gastrointestinal release [78].

Despite these advantages, CD-based systems present limitations that may hinder broader clinical translation. High CD concentrations can increase formulation bulk and, in some cases, induce osmotic or gastrointestinal tolerance issues. β-CD carries known nephrotoxicity risks at elevated doses, whereas HP-β-CD, though safer, may still suffer from limited loading capacity for highly lipophilic flavonolignans. Additionally, inclusion complexes alone may not fully resolve silymarin’s extensive first-pass metabolism, necessitating combination strategies with permeability enhancers or nano-carriers. Overall, CD-based inclusion complexes represent a robust and scalable approach to improving the biopharmaceutical performance of silymarin. However, future development should focus on optimizing drug-CD stoichiometry, minimizing CD-associated excipient burden, and integrating inclusion with advanced delivery systems to address both solubility and metabolic barriers.

### 4.3. Particle Size Reduction

Particle size reduction is a widely employed strategy to enhance the dissolution rate and oral bioavailability of poorly water-soluble compounds like silymarin. According to the Noyes–Whitney equation, decreasing particle size increases surface area and dissolution velocity, thereby facilitating faster drug absorption across gastrointestinal membranes [70].

Two principal methodologies are utilized for particle size reduction: (i) Top-down techniques, including mechanical processes such as media milling and high-pressure homogenization, physically break down larger drug particles into nanoscale dimensions. (ii) Bottom-up techniques, such as antisolvent precipitation and solvent evaporation, promote the nucleation and growth of nanocrystals or amorphous nanoparticles from drug solutions under controlled conditions [92].

Bottom-up approaches are particularly attractive due to their operational simplicity, scalability, and relatively low cost. For instance, Sahibzada et al. developed an amorphous nanosuspension of silymarin using precipitation methods, which exhibited improved solubility and enhanced antibacterial activity compared to raw drug formulations [70]. In another study, Jabeen et al. utilized antisolvent precipitation to generate silymarin nanocrystals, which were subsequently embedded into a chondroitin sulfate-based thermoresponsive hydrogel. The bioavailability was 2-fold higher than silymarin suspension. This formulation also achieved sustained drug release over 48 h, offering potential for long-acting therapeutic applications [93].

Overall, particle size reduction technologies present a robust strategy for overcoming solubility and permeability barriers, particularly when integrated with stabilizing carriers or gels, laying the groundwork for improved silymarin delivery in both conventional and extended-release formats.

### 4.4. Lipid-Based Carriers

Lipid-based formulations have emerged as one of the most effective strategies to improve the oral bioavailability, chemical stability, and therapeutic efficacy of silymarin and silybin. These systems leverage the natural lipid absorption pathways in the gastrointestinal tract, facilitating lymphatic transport and partially bypassing hepatic first-pass metabolism. Common lipid-based delivery systems include phospholipid complexes (phytosomes), liposome, nanoemulsions, self-microemulsifying drug delivery systems (SMEDDSs), and lipid nanoparticles. Comparative pharmacokinetic studies have shown that these systems can achieve 2.5- to 9.6-fold improvements in oral bioavailability relative to conventional silymarin tablets such as Legalon^®^ [62,75]. In some cases, nanostructured lipid systems have exhibited 70- to 80-fold increases in in vitro dissolution rates, translating into significantly improved systemic exposure and therapeutic outcomes [94].

#### 4.4.1. Phytosomes

Silybin exhibits poor membrane permeability due to its relatively large molecular weight and hydrophilic nature, which severely limits its ability to passively diffuse across intestinal epithelial membranes [69]. Phytosomes, a class of lipid-based vesicular complexes, address this barrier by conjugating silybin with phospholipids (typically phosphatidylcholine), thereby enhancing both its lipophilicity and membrane affinity [95,96]. This complexation generally occurs through hydrogen bonding between the hydroxyl groups of silymarin and the polar head groups of the phospholipids, forming amphiphilic molecular assemblies with improved biopharmaceutical properties.

Following oral administration, the enzymatic digestion of phosphatidylcholine generates liquid crystalline intermediates such as micelles and vesicles, which facilitate intestinal transport and absorption [95,97]. For instance, silymarin-loaded phytosomes (~100 nm) prepared via the thin-film hydration method showed significant therapeutic efficacy in models of alcoholic liver disease [98]. In a clinical pharmacokinetic study, a silymarin-phosphatidylcholine soft complex achieved a 9.6-fold increase in oral bioavailability compared to conventional silymarin tablets in healthy volunteers [62].

Additionally, phytosomal formulations have demonstrated promising results in oncology settings. Compared to other formulations such as silybin-meglumine and Eurosil 85, phospholipid complexes exhibited superior intestinal absorption, blood–brain barrier (BBB) penetration, and overall clinical efficacy in patients with advanced systemic cancers and brain metastases [69].

#### 4.4.2. Liposomes

Liposomes are spherical vesicular carriers composed of one or more phospholipid bilayers surrounding an aqueous core, often stabilized with cholesterol. This unique architecture allows them to encapsulate both hydrophilic drugs (within the aqueous core) and lipophilic drugs (within the lipid bilayer), making them highly versatile for drug delivery. In the context of silymarin, liposomes offer multiple pharmacological advantages, including enhanced solubility, protection against enzymatic degradation, and tissue-specific targeting [99,100].

Silymarin-loaded liposomes have demonstrated potent antioxidant and anti-apoptotic properties in vitro. In cell culture studies using MRC-5 and A549 cell lines, these formulations significantly improved cell viability by reducing reactive oxygen species (ROS) and downregulating pro-apoptotic markers such as cleaved poly ADP-ribose polymerase (PARP) and caspase-3 [101]. To further enhance delivery efficiency and pharmacokinetic performance, various surface-modified liposomes have been explored. Notably, PEGylated liposomes showed markedly improved systemic exposure, with a 4-fold increase in AUC compared to conventional silymarin suspensions. Similarly, hexadecyl phosphate- and stearamine-modified liposomes exhibited improved hepatic accumulation and therapeutic efficacy [102].

Despite these benefits, liposomes are inherently prone to physical instability, including vesicle aggregation, drug leakage, and lipid oxidation. To address these limitations, proliposome formulations have been developed. These dry, free-flowing powders spontaneously convert into liposomes (<100 nm) upon hydration, offering enhanced stability and ease of handling. In vivo studies demonstrated that proliposomes achieved a 2-fold higher AUC and significantly reduced liver histopathology compared to conventional silymarin tablets [103].

#### 4.4.3. Nanoemulsions and SMEDDSs

Nanoemulsions are kinetically stable colloidal systems composed of oil, water, surfactants, and occasionally co-surfactants, with droplet sizes typically ranging from 20 to 200 nm. Their small size leads to an increased interfacial surface area and enhanced mucosal adhesion, facilitating solubilization and intestinal absorption of lipophilic drugs such as silymarin. In preclinical models, silymarin nanoemulsions demonstrated improved cardioprotective effects in 5-fluorouracil-induced cardiotoxicity and enhanced brain delivery following intranasal administration in a Parkinson’s disease model, attributed to improved mucosal permeability and central nervous system bioavailability [104,105]. However, long-term physical stability remains a challenge, with risks of droplet coalescence, phase separation, and sedimentation during storage.

SMEDDSs are isotropic mixtures of oil, surfactants, and co-solvents that spontaneously form fine oil-in-water microemulsions (<100 nm) upon contact with gastrointestinal fluids. These systems maintain the drug in a solubilized state and enhance lymphatic transport, partially bypassing hepatic first-pass metabolism via chylomicron stimulation [87,106]. Compared to conventional solid dispersions, cyclodextrin complexes, and marketed formulations like Legalon^®^, silymarin-loaded SMEDDSs significantly improved solubility, dissolution rate, and systemic exposure [76].

To mitigate drug precipitation under gastric conditions, supersaturable SMEDDSs (SSMEDDSs) have been developed by incorporating precipitation inhibitors (e.g., Poloxamer 407), enabling the maintenance of supersaturation in vivo. These formulations achieved a 7.6-fold increase in oral bioavailability compared to Legalon^®^ [74]. Moreover, pH-sensitive SMEDDSs (pH-SMEDDSs) have been engineered to modulate drug release in response to gastrointestinal pH changes, resulting in a 70- to 80-fold enhancement in in vitro dissolution and superior hepatoprotective effects in carbon tetrachloride-induced liver injury models [94].

#### 4.4.4. Lipid Nanoparticles

Lipid nanoparticles (LNPs) represent a next-generation oral delivery platform that mimics the physiological digestion and absorption pathways of dietary lipids. By stimulating bile secretion and facilitating the formation of mixed micelles, LNPs enhance the solubility, stability, and intestinal uptake of lipophilic compounds such as silymarin [103,107]. These systems are generally categorized into three types: solid lipid nanoparticles (SLNs), nanostructured lipid carriers (NLCs), and lipid-drug conjugates (LDCs) [108,109,110].

SLNs are composed of biocompatible solid lipids and provide sustained drug release and enzymatic protection. However, their crystalline nature can limit drug loading. In contrast, NLCs incorporate a mixture of solid and liquid lipids to prevent crystallization, enhance drug encapsulation efficiency, and improve release profiles [75,111,112,113]. In one research of Wei Wu group, silymarin-loaded NLCs prepared via high-pressure homogenization demonstrated a 2.54-fold increase in oral bioavailability compared to Legalon^®^ and a 3.10-fold increase relative to solid dispersions in beagle dog models [75]. Pharmacokinetic data further revealed that SLNs exhibited slower degradation and prolonged plasma retention, likely due to gradual enzymatic breakdown, resulting in superior bioavailability compared to NLCs and fast-release formulations [108].

LDCs represent an emerging strategy in which drug molecules are covalently linked to fatty acids, creating prodrug-like systems with improved lipophilicity and metabolic stability. Fatty acid–silybin conjugates (FA-SBs) synthesized with varying chain lengths (C6, C12, C18) revealed that C12-linked LDCs offered the optimal balance of oral stability and systemic exposure [79].

In vivo, silybin-loaded LNPs effectively alleviated hyperglycemia, dyslipidemia, and peripheral neuropathy in streptozotocin-induced diabetic mice [112]. Moreover, brain concentrations of silymarin were increased by 12.46-fold, and behavioral tests demonstrated antidepressant effects comparable to fluoxetine, suggesting promising central nervous system targeting capabilities [114].

**Figure 5:**
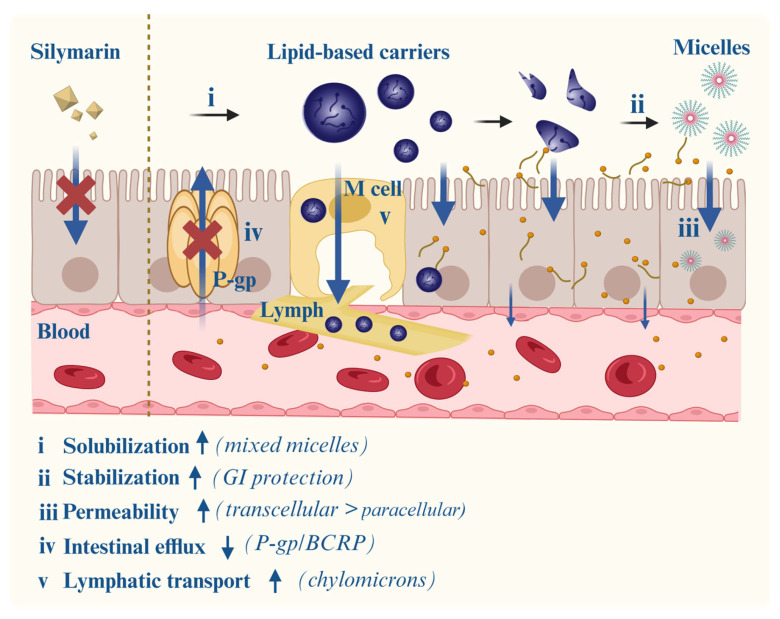
Lipid-based carriers (e.g., phytosomes, liposomes, nanoemulsions, SMEDDSs, and lipid nanoparticles) enhance the oral absorption of silymarin through multiple mechanisms: (i) solubilizing poorly water-soluble flavonolignans; (ii) protecting against gastrointestinal degradation; (iii) improving membrane permeability via close interaction with enterocytes; (iv) inhibiting efflux transporters such as P-gp and BCRP; and (v) promoting lymphatic uptake to bypass hepatic first-pass metabolism. Together, these processes synergistically improve systemic exposure and therapeutic efficacy. Note: self-microemulsifying drug delivery systems (SMEDDSs); gastrointestinal (GI); P-glycoprotein (P-gp); breast cancer resistance protein (BCRP). ↑ means increased and ↓ means decreased.

In summary, lipid-based carriers have emerged as versatile platforms to overcome the biopharmaceutical barriers of silymarin. Their effectiveness arises from multiple synergistic mechanisms (illustrated in Figure 5): enhancing solubilization, improving intestinal permeability, promoting lymphatic transport to bypass first-pass hepatic metabolism, and in some cases inhibiting efflux transporters. By integrating these complementary pathways, lipid carriers represent one of the most clinically relevant strategies for improving the oral absorption and therapeutic consistency of silymarin.

### 4.5. Polymeric Micelles

Polymeric micelles are nanoscale colloidal carriers formed through the self-assembly of amphiphilic block copolymers in aqueous environments. These structures consist of a hydrophobic core, which solubilizes poorly water-soluble drugs like silymarin, and a hydrophilic corona, which stabilizes the micelles, enhances gastrointestinal transit, and protects the encapsulated drug from enzymatic degradation [115,116].

Silymarin-loaded polymeric micelles have demonstrated significant improvements in oral bioavailability, enhanced transmembrane permeability, and organ-protective effects in preclinical models. For instance, micellar formulations mitigated cisplatin-induced nephrotoxicity, suggesting a potential role in adjunct therapy [117].

To improve micellar stability and overcome the inherent limitations of conventional polymeric systems (e.g., low drug loading and high critical micelle concentration [CMC]), mixed micelle systems have been developed. A formulation based on HS15-Poloxamer 188 increased silymarin’s dissolution rate by 460-fold, while also enhancing antioxidant capacity and neuroprotective effects in oxidative stress models [118]. Further innovation has led to hybrid polymeric micelles engineered for liver-targeted delivery. For example, hyaluronic acid-based micelles co-conjugated with glycyrrhizic acid (a hepatotropic ligand) and deoxycholic acid achieved improved hepatic biodistribution and bioactivity [119]. These systems exploit ligand-receptor interactions and bile acid transport mechanisms to facilitate site-specific delivery.

Despite these advances, polymeric micelles still face limitations, such as premature drug release, relatively low encapsulation efficiency, and potential instability under physiological conditions. However, mixed micelle and hybrid strategies have partially addressed these issues by enhancing drug retention, sustained release, and pharmacological performance [120,121,122] (Table 3).

## 5. Molecular Modification and Bioavailability Enhancers

Lipid-based and nanocarrier systems have demonstrated remarkable success in enhancing the oral bioavailability of silymarin. However, these approaches can be constrained by formulation complexity, stability issues, and manufacturing costs. To complement such delivery systems, molecular-level strategies have been explored, including chemical derivatization and co-administration with bioavailability enhancers (BEs). These methods aim to improve aqueous solubility, metabolic stability, and intestinal permeability of silymarin through relatively simple and scalable interventions, making them attractive for clinical translation and fixed-dose combinations.

This section highlights two major directions: (i) the development of water-soluble salts and structural derivatives of silymarin to improve physicochemical and pharmacokinetic profiles, and (ii) the application of natural and synthetic BEs to modulate enzymatic and transporter-mediated barriers.

### 5.1. Water-Soluble Salts and Derivative Methods

**Figure 6:**
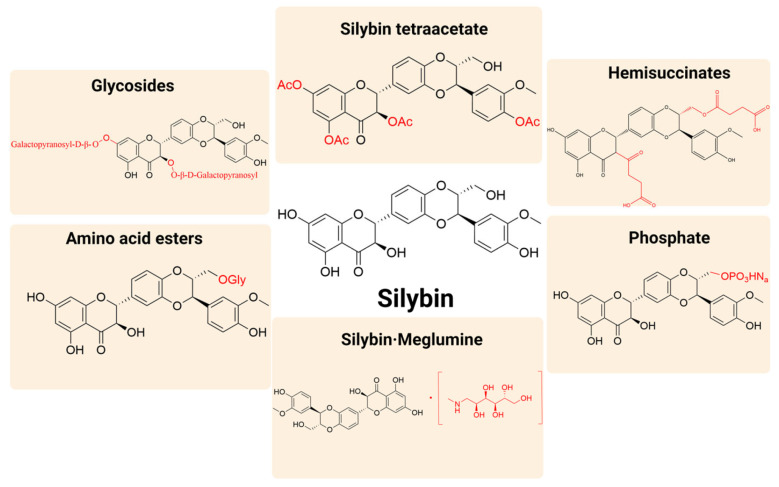
Water-soluble salts and derivatives of silybin. The core structure (parent scaffold) of silybin is highlighted in black, while the various modifying groups or functional groups introduced at different positions to form salts or derivatives are shown in red.

Direct molecular modification provides a streamlined strategy to overcome silymarin’s biopharmaceutical limitations, complementary to lipid-based or nanocarrier systems. Among these strategies, water-soluble salts and structural derivatives (such as tetraacetates, glycosides, amino acid esters, phosphates, hemisuccinates, and silybin meglumine) have shown particular promise in enhancing aqueous solubility, improving metabolic stability, and expanding formulation versatility (Figure 6).

Silybin meglumine provides a marked improvement in aqueous solubility compared with free silybin, enabling its incorporation into both oral and parenteral formulations. In vitro, it shows hepatoprotective effects and antiproliferative activity against cancer cells [78]. However, despite this relative enhancement, the solubility of the meglumine salt remains insufficient for high-concentration injectable use, and the compound shows rapid systemic clearance, limiting sustained plasma exposure. These drawbacks have motivated the development of alternative strategies, including mesoporous silica carriers [77] and orally disintegrating tablets (ODTs) [92,126,127], to improve stability, prolong exposure, and achieve more flexible dosing. A representative silymarin-based ODT achieved disintegration within 12.5 s and demonstrated protective effects in a pulmonary toxicity model [126]. Additional derivatives, such as glycol-conjugates, are being explored for improved formulation stability and bioavailability [128].

Comparative pharmacokinetic studies show that silybin meglumine achieves up to a 30-fold increase in oral bioavailability, which exceeds improvements seen with cyclodextrin complexes (20-fold), phospholipid complexes (10-fold), and solid dispersions (5-fold) [30]. Importantly, these enhancements arise from direct molecular modification rather than incorporation into nanoparticle systems, addressing potential confusion in earlier citations.

### 5.2. Bioavailability Enhancers

BEs are adjunct agents that improve systemic drug exposure by increasing absorption, inhibiting first-pass metabolism, or modulating efflux transporters. Natural BEs, such as piperine (PIP) from Piper species and Zingiber officinale, have long been used in traditional medicine to potentiate herbal remedies [129,130]. PIP enhances oral bioavailability by inhibiting CYP3A4/5, blocking intestinal efflux transporters (P-gp, BCRP), and increasing epithelial permeability [131,132,133]. As silymarin is a substrate of P-gp and BCRP, co-administration with PIP significantly improves absorption and systemic exposure [134], with animal studies showing a 146–181% increase in oral bioavailability [135].

Some studies have evaluated BE-loaded nanoparticle systems, where BEs amplify the performance of an existing nanocarrier. These examples illustrate synergistic enhancement, not the intrinsic simplicity or scalability of nanoparticles. To avoid confusion, we now explicitly clarify that BEs as standalone molecular additives, rather than BE-loaded nanocarriers, represent strategies that are typically simpler, more scalable, and easier to integrate into conventional dosage forms. For example, solid dispersions with D-α-TPGS increased silymarin solubility by 23-fold and improved hepatic exposure 3.4-fold without requiring nanoparticle fabrication [35]. Similarly, solubilizers such as PEG 400, Pluronic P85/F127, and Tween^®^ 80 improve permeability through well-established, GMP-compatible mechanisms [135].

In summary, molecular modification (salts, derivatives) are simpler and more manufacturing-feasible than most nanosystems. When BEs are integrated into nanoparticles, their role should be viewed as synergistic augmentation rather than evidence that BE-based approaches require complex carriers. We have revised the section to clearly distinguish these two concepts.

## 6. Non-Oral Delivery Strategies for Silymarin

Despite significant progress in oral delivery technologies, these approaches remain inherently constrained by the gastrointestinal tract. For patients with hepatic insufficiency, gastrointestinal disfunction, swallowing difficulties, or conditions requiring rapid systemic exposure, oral formulations (even when highly optimized), may still prove inadequate. In such cases, non-oral delivery routes offer valuable alternatives, enabling the circumvention of first-pass metabolism, improving dose precision, and supporting localized or sustained drug exposure. This section summarizes current advances in parenteral, transdermal/dermal, and controlled-release devices and evaluates their potential to further extend the therapeutic reach of silymarin.

### 6.1. Targeted Parenteral Delivery

Parenteral administration offers immediate systemic exposure and precise dose control, making it ideal for critical conditions where oral bioavailability is insufficient. As discussed in previous sections, salt formation (e.g., silybin meglumine) substantially improves the aqueous solubility of silybin and has been successfully incorporated into oral preparations. Building on these solubility advantages, early clinical and preclinical studies also evaluated silybin meglumine in injectable forms. However, when transitioned from the gastrointestinal environment to the parenteral route, this salt exhibits several critical limitations. Its solubilization relies on highly alkaline pH conditions, and the solution is prone to precipitation at physiological pH, which severely restricts its compatibility with intravenous administration. Moreover, the formulation shows limited chemical and physical stability, further constraining its clinical utility. Thus, although salt formation markedly enhances intrinsic solubility, it does not provide the robust, physiologically compatible solubility required for parenteral delivery, underscoring the need for more sophisticated nanoparticle-based injectable systems.

To address these issues, nanoparticle-based injectable systems have gained attention. These carriers not only enhance solubility and stabilize silymarin under physiological conditions but also prolong systemic circulation, reduce nonspecific distribution, and permit passive or ligand-mediated targeting. A notable example is human serum albumin (HSA)-based nanoparticles loaded with silybin-phospholipid complexes. These nanoparticles leverage HSA’s biocompatibility and natural hepatic affinity to selectively accumulate in liver tissue following intravenous injection, offering a promising strategy for liver-targeted therapy, including hepatocellular carcinoma treatment [81].

Additionally, cholesterol/1,2-distearoyl-sn-glycero-3-phosphoethanolamine (DSPE)-coated nanobubbles, capable of encapsulating both hydrophobic drugs and gas, have been developed for intravenous co-delivery of silybin and camptothecin in ovarian cancer therapy. Galactose-functionalized hyaluronic acid (Gal-HA) coated cationic solid lipid nanoparticles carrying silybin enabled specific liver surface targeting of asialoglycoprotein receptor (ASGPR) and cluster of differentiation 44 (CD44) proteins to enhance silybin uptake to achieve accurate medication for alcoholic liver injury [136]. This dual-drug system demonstrated enhanced anticancer efficacy, tumor specificity, and reduced systemic toxicity [137], illustrating the potential of combinatorial nanocarrier platforms in complex diseases.

### 6.2. Topical, Dermal and Transdermal Delivery

In addition to systemic delivery, localized non-oral systems, including topical, dermal, and transdermal formulations, have attracted growing interest for exploiting silymarin’s anti-inflammatory, antioxidant, and photoprotective properties [138,139]. Silymarin and its key constituent silybin protect against UVA-induced oxidative stress in human dermal fibroblasts, while minor flavonolignans such as 2,3-dehydrosilybin and silychristin offer particularly strong UVA-protective effects [138]. Based on this pharmacological profile, silymarin has been studied in a range of dermatological conditions, including vitiligo [139], acne [140], burns [141], and melasma [63,142,143,144,145]. However, its low water solubility and limited skin permeability restrict clinical efficacy in topical applications.

To overcome these barriers, advanced nanocarrier systems have been employed: (i) Polymeric micelles enhanced drug accumulation in depigmented areas, showing efficacy in vitiligo treatment [139]. (ii) Nanogels improved photostability, provided sustained release, and demonstrated excellent dermal compatibility [146,147]. (iii) Chitosan-coated vesicles significantly increased transdermal penetration and showed promise in wound healing [148]. (iv) Electrospun nanofiber mats based on polycaprolactone loaded with silymarin accelerated wound closure and served as bioactive dressings in animal models [149]. (v) Mucosal-targeted in situ gels containing silymarin-loaded NLCs enabled prolonged residence at the oral mucosa, offering localized anticancer potential for oral lesions [150]. These innovations expand silymarin’s therapeutic reach beyond hepatic diseases, supporting its topical and transmucosal application in skin and oral pathologies.

### 6.3. Controlled-Release of Silymarin

Silymarin’s short half-life, poor gastrointestinal stability, and low bioavailability often require frequent dosing, which may compromise therapeutic outcomes and reduce patient adherence. To address this, controlled-release systems have been developed to prolong systemic exposure, reduce dosing frequency, and stabilize plasma concentrations.

Among these, molecularly imprinted polymers (MIPs) offer tailored, diffusion-controlled release. Ji et al. designed hollow magnetic MIPs capable of sustaining silymarin release for up to 36 h in simulated gastric conditions [151], indicating their potential for long-acting oral or implantable applications. Thermosensitive hydrogels, such as chondroitin sulfate-based matrices loaded with silymarin nanocrystals, have achieved 48-h sustained release [93], representing a patient-friendly, minimally invasive delivery route.

**Figure 7:**
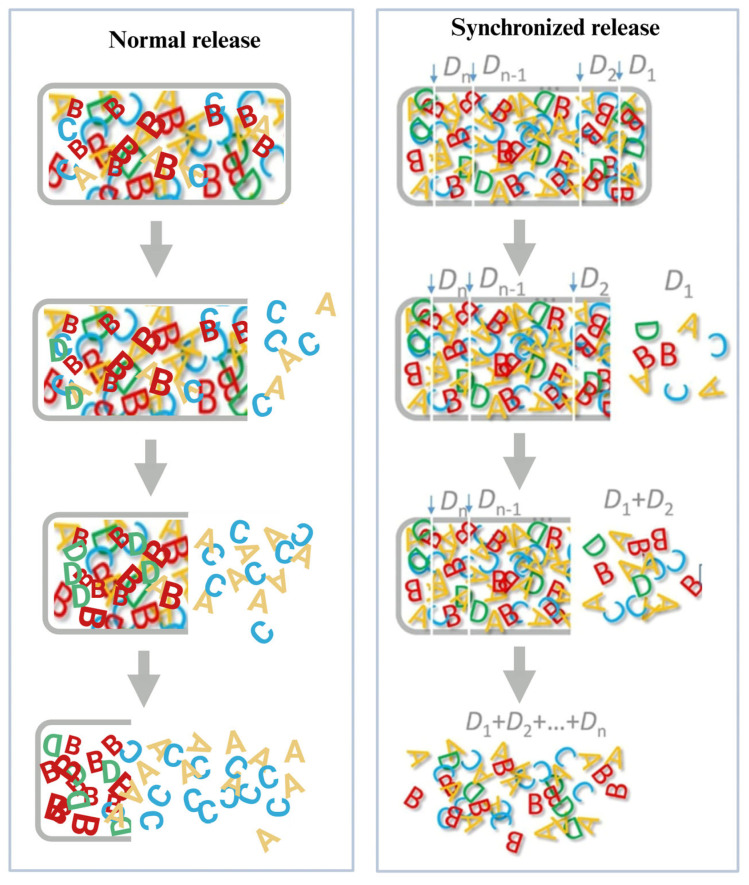
Infinite dose dividing, a solution for synchronized release [152]. This schematic contrasts release strategies for silymarin constituents (A–D). The novel “infinite dose dividing” approach packages the total dose into numerous micro-units (D_1_–D_n_), each containing all constituents. These units release rapidly and in synchrony as intact entities, preserving the native ratio. In contrast, conventional systems (“Normal Release”) often exhibit differential and asynchronous release due to the varying physicochemical properties of the components, which may compromise therapeutic synergy.

However, the multi-component nature of silymarin poses unique challenges. Its therapeutic effects derive from synergistic ratios of flavonolignans, such as silybin, isosilybin, and silychristin. Conventional sustained-release systems may unevenly release these constituents due to their differing physicochemical properties, potentially disrupting pharmacodynamic synergy. To overcome this, a novel strategy of synchronous release has been proposed (illustrated in Figure 7). This concept maintains the native component ratio over time by dividing the total dose into multiple microdoses (D_1_–D_n_), each preserving the original composition. These are sequentially released via pH-responsive matrices, erosion-controlled carriers, or osmotic pump systems, maintaining pharmacological balance throughout the dosing interval [152]. Our group developed an erodible matrix system using glycerol monostearate and PEG 6000 or poloxamer 188, achieving highly similar release curves across all constituents, thus validating the synchronous release model [153].

Additional platforms demonstrating this approach include: (i) Solid dispersion dropping pills, enhancing solubility and achieving synchronized release of all major flavonolignans [154]. (ii) Microporous osmotic pump tablets, offering zero-order, 12-h release with 85% cumulative delivery [127]. (iii) Single-chamber osmotic pump tablets, enabling synchronized, zero-order release for up to 20 h [155]. These technologies represent a promising direction for delivering multi-component phytopharmaceuticals like silymarin with precision and consistency.

## 7. Translational and Regulatory Barriers to Clinical Commercialization of Advanced Silymarin Delivery Systems

Despite decades of promising laboratory research, including extensive development of nano-enabled formulations, the global silymarin market remains dominated by conventional oral products such as standardized extracts, dispersible tablets, dripping pills, and salification-based derivatives (details list in Table 2). The contrast between abundant preclinical innovation and the absence of clinically approved advanced systems underscores a persistent “translation gap.” Notably, even the few improved products that reached the market (silybin-phospholipid complexes and injectable silybin succinate) represent incremental enhancements rather than truly advanced nanocarriers, and both continue to face manufacturing, stability, and regulatory challenges [62,66]. This real-world experience provides important context for understanding why most cutting-edge delivery systems fail to progress beyond the experimental stage.

### 7.1. Why Nanoparticle-Based Silymarin Systems Have Not Reached Clinical Trials

Although hundreds of studies describe liposomes, nanoemulsions, polymeric nanoparticles, SLNs/NLCs, and micellar systems for silymarin and silybin, no nanotechnology-enabled silymarin formulation has yet progressed into regulated clinical development. To date, the only next-generation system to enter a registered human study is the micellar formulation LipoMicel^®^ (NCT06882681, initiated in 2024), a small bioavailability trial whose results have not been released. Beyond this isolated example, nanoparticle-based systems remain absent from Phase I clinical pipelines across major registries (ClinicalTrials.gov). This absence reflects several key barriers:

(i) Manufacturing and scale-up limitations. Most nanoparticle systems reported in academia rely on solvent evaporation, nanoprecipitation, sonication, or microemulsion methods that show inability to maintain particle size, PDI, and encapsulation efficiency at scale; batch-to-batch variability incompatible with GMP; difficulty achieving stability metadata required for investigational new drug (IND) submission (e.g., ≥24-month room-temperature stability) [156].
(ii) Regulatory CMC complexity for natural-product nanomedicines. Nanomedicines require stringent physicochemical characterization (size distribution, surface chemistry, release profile, impurity analysis). For plant-derived mixtures like silymarin, additional challenges include multi-constituent variability; need to characterize active pharmaceutical ingredient (API) distribution inside nanostructures; unclear classification (drug vs. herbal drug vs. complex mixture vs. nanomedicine); These burdens often exceed the capacity of academic-industry teams in early-phase development [156,157].
(iii) Lack of clear clinical differentiation. To justify the cost and regulatory burden of nanomedicines, new formulations must outperform established oral complexes (e.g., Legalon^®^). Most nanoformulations report improved PK in rodents but lack disease-relevant efficacy models; comparative superiority to existing prescription standards; well-defined clinical targets and endpoints. As a result, they cannot support IND applications for first-in-human trials [158].

### 7.2. Barriers Extending to Parenteral, Transdermal, and Controlled-Release Systems

The translational bottlenecks observed for nanoparticle systems also apply to other non-oral routes:

(i) Parenteral formulations. Although intravenous injection theoretically circumvents solubility and first-pass metabolism, real-world experience with silybin meglumine and silybin succinate injectables reveals substantial constraints. These include solubility achievable only under extreme alkaline pH; precipitation risk after dilution or during infusion; sensitivity to light, temperature, and handling; limited long-term physicochemical stability. These issues underscore that parenteral delivery does not automatically solve silybin’s biopharmaceutical limitations and may even amplify development risks associated with nano-injectables [81].
(ii) Transdermal patches and microneedle systems. Preclinical benefits (enhanced permeation, bypassing first-pass metabolism) have not translated due to large interspecies differences in stratum corneum permeability, complicating pharmacokinetics extrapolation; stricter biocompatibility requirements for skin-contact materials; lack of a clinically validated transdermal indication, as hepatoprotective therapy is inherently systemic and traditionally oral.
(iii) Controlled-release implants or oral depot systems. These platforms require clear therapeutic rationales (e.g., prolonged stable plasma levels), yet most silymarin indications do not demand depot delivery. The complexity of CMC controls for release kinetics and device-drug integration further discourages progression into clinical development.

### 7.3. Principles for Future Successful Translation

The developmental history of silymarin delivery systems indicates that technological sophistication alone is insufficient for clinical success. Based on current evidence and the translational challenges discussed above, several practical considerations may support the development of clinically viable next-generation formulations:

(i) Clearly define the clinical rationale for advanced delivery. Target indications where advanced delivery offers a clear advantage, such as acute hepatotoxicity (requiring rapid exposure), chronic liver disease (needing sustained levels), or oncology (leveraging enhanced permeability and retention).
(ii) Consider manufacturability and stability at an early stage. Formulation design should prioritize processes that can be feasibly scaled, minimize reliance on organic solvents, and maintain key quality attributes such as particle size and chemical stability. Early establishment of stability programs aligned with internationally recognized quality guidelines can reduce downstream development delays.
(iii) Engage proactively with regulatory frameworks. Engage early with regulatory agencies (EMA/FDA) to clarify pathways for nanomedicines and/or botanical drug products.
(iv) Demonstrate clinically relevant advantages over existing products. Demonstrate not just improved pharmacokinetics, but clinically meaningful efficacy advantages over the current standard of care (e.g., silybin-phospholipid complexes).

By systematically addressing these intertwined scientific, regulatory, and commercial barriers, the field can begin to bridge the long-standing gap between promising preclinical formulations and clinically impactful, commercially viable silymarin therapies.

## 8. Conclusions and Perspectives

Building on centuries of traditional use and decades of pharmacological exploration, silymarin has emerged as a representative model for modernizing complex botanical medicines. Silymarin, a traditional polyphenolic extract from *Silybum marianum*, exhibits well-documented antioxidant, hepatoprotective, anti-inflammatory, and anticancer activities. This review has comprehensively examined its historical usage, pharmacological actions, clinical applications, and, most critically, its biopharmaceutical limitations. Despite its multifaceted therapeutic potential, the clinical performance of silymarin is hampered by poor oral bioavailability due to low aqueous solubility, hydrophobicity, enzymatic degradation, limited intestinal absorption, and rapid systemic clearance.

To address these challenges, multiple formulation strategies have been developed: (i) solid dispersions, salt/derivative modification, and particle size reduction to improve solubility and dissolution; (ii) bioavailability enhancers to inhibit efflux transporters and first-pass metabolism; (iii) lipid-based carriers to facilitate membrane transport and lymphatic uptake, including phospholipid complexes, SMEDDSs, and SLNs; and (iv) non-oral delivery routes for bypassing gastrointestinal barriers and achieving targeted or sustained drug action, including nanoparticulate injectables, transdermal patches, and controlled-release platforms. Importantly, silymarin’s therapeutic efficacy depends on the coordinated action of multiple flavonolignan components. This poses unique challenges for sustained-release formulations, which often lead to asynchronous release and pharmacodynamic inconsistency. In response, this review introduces the concept of synchronous release and infinite dose allocation, aiming to preserve the stoichiometric and temporal integrity of multicomponent phytomedicines. Beyond laboratory advances, we further evaluated currently marketed silymarin preparations and recent clinical/preclinical findings. Despite decades of research, there remains a striking gap between high-performance experimental systems and the limited range of formulations available in clinical practice (primarily simple tablets, capsules, and phospholipid complexes). Our analysis identifies key translational bottlenecks, including manufacturing scalability, excipient safety, regulatory categorization of botanical/nanotechnology hybrids, and the need for harmonized quality standards.

Looking forward, bridging this bench-to-bedside disconnect will require drug-delivery platforms that balance biopharmaceutical performance, manufacturability, regulatory compatibility, and clinical practicality. Integrating nanotechnology with smart excipients, scalable processing, and patient-centric design offers a promising pathway toward clinically deployable, next-generation silymarin formulations. More broadly, the framework proposed in this review, centering on mechanistic understanding, coordinated multi-component delivery, and translational feasibility, may serve as a generalizable model for the modernization of complex botanical medicines in the era of precision phytotherapy.

## Figures and Tables

**Table 1:** Representative clinical trials of silymarin/silybin formulations.

Formulation Type	Study ID/Year	Dose & Regimen	Disease/Indication	Primary Outcomes	Reference(s)
Conventional standardized extract; (Silymarin/Legalon^®^)	NCT00680407 (2008)	Oral; 420 or 700 mg Tid	Non-alcoholic steatohepatitis	No significant histological benefit; High dose safety established	[59]
	NCT01752153 (2012)	Oral; 40 mg/Kg/day + Desferrioxamine	Immune abnormalities	—	—
	NCT04394208 (2020)	Oral; 140 mg Tid	COVID-19 pneumonia	—	[43]
	NCT05631041 (2022)	Oral; 140 mg Qd	Metastatic colorectal cancer	—	—
IV; Silibinin (Legalon-SIL^®^)	NCT01518933 (2011)	IV; 20 mg/kg daily, 2-h infusion × 14 days	Hepatitis C virus recurrence	Viral load reduced by 2.30 ± 1.32 log in silibinin group vs. no change in placebo (*p* = 0.0002)	[60]
Two different milk thistle formulations	NCT02529605 (2015)	Product B^®^ vs. IsaGenesis^®^ (crossover design)	Healthy; bioavailability study	Dose-corrected C_max_ increased 365% (silybin A) and 450% (silybin B) vs. powdered form	[61]
Silibin-Phytosome	NCT00487721 (2007)	Oral; 13 g/day	Prostate cancer	—	—
Phospholipid complex (Siliphos^®^)	NCT01129570 (2010)	Oral; 2–12 g/day	Advanced hepatocellular carcinoma	—	—
Silibinin-phosphatidylcholine complex	NCT03440164 (2016)	Oral; 45 mg silibinin soft-gel vs. 70 mg standard silymarin tablets	Healthy; Bioavailability study	AUC_0–∞_: soft-gel 308.8 ± 126.1 vs. tablets 29.5 ± 14.1 ng·h/mL (*p* < 0.0001)	[62]
Nutraceutical blend (Vitamin E + Silymarin + Carnitine)	NCT01511523 (2012)	Oral combination	NAFLD	—	—
Silymarin 1.4% cream	NCT04490967 (2021)	Topical; Bid	*Acne vulgaris*	—	—
Silymarin 0.7–1.4% cream	NCT03982849 (2019)	Topical; 0.7–1.4% cream vs. 4% hydroquinone; 3 months	Melasma	All reduced MASI score; no significant difference between groups	[63]
Silymarin topical + microneedling	NCT05099601 (2021)	Topical; Silymarin 0.7% alone vs. combined with microneedles	Melasma	—	—
Micellar silymarin (LipoMicel^®^ soft-gel)	NCT06882681 (2024)	Oral; ~140 mg micellar silymarin/capsule vs. standard silymarin extract	Healthy; bioavailability study	—	—

Note: — indicates data are no reported. Melasma area and severity index (MASI), area under curve (AUC).

**Table 3:** Summary of representative formulation strategies for improving silymarin or silybin delivery.

Delivery Strategy	Key Composition/Materials	Intended Biopharmaceutical Improvement	Main Findings	Limitations/Translational Barriers	Reference(s)
Solid Dispersions	Silymarin; Tween 80; HPC	Improve solubility, dissolution, absorption	Relative bioavailability ↑ 215–589%	Stability issues; moisture sensitivity	[87]
	Kollidone VA64; Soluplus; Poloxamer 188	Increase dissolution rate, amorphization	Release ↑ 87.8%/120 min; stability constant ↑ 2.6–5.3×	Polymer load high; scale-up challenges	[88]
	Silymarin; TPGS	Solubility ↑; permeability ↑; efflux inhibition	Solubility ↑ 23×; absorption ↑ 4.6×; liver distribution ↑ 3.4×; efflux ratio ↓	Surfactant-related toxicity concerns	[35]
	Silymarin; PVP	Improve dissolution and absorption	Relative bioavailability ↑ 5× in dogs	Polymer recrystallization risk	[80,86]
Cyclodextrin Systems	Silymarin; β-CD; TPGS	Solubility enhancement; release modulation	Release ↑ 99%/45 min; IC50 ↓	Large CD dose; GI tolerability concerns	[89]
	Silymarin; HP-β-CD; erythritol	Solubilization via complexation	Release ↑ 80%/60 min	CD complexation efficiency variable	[123]
	Silymarin; β-CD; Zein	Solubility ↑; sustained release	EE 84%; Bioavailability ↑	Hybrid NP-CD system → classification/regulatory ambiguity	[124]
Lyophilized Nanosuspension Tablets	Silymarin; PVA; mannitol	Nanosizing → dissolution ↑	Size 277 nm; disintegration < 30 s	Stability during storage; aggregation	[92]
Nanocrystal Hydrogels	Silymarin; chondroitin sulfate	Solubility ↑; sustained release	Release ↑ 89%/24 h; bioavailability ↑ 2×	Limited scalability; gel rheology variability	[93]
Phytosomes/Phospholipid Complexes	Silybin–phosphatidylcholine	Membrane permeability ↑	Bioavailability ↑ 9.6×	High phospholipid cost; variability	[62]
	Silybin–PC	BBB permeability improvement	Papp 6.29 × 10^−6^ cm/s	Limited clinical data	[69]
	Silymarin–PC	Enhanced hepatoprotection	Bioavailability ↑ 6×; size 218 nm	Component variability	[95]
	Silymarin phytosome NPs	Protection from inflammation	Size~100 nm; reduced ethanol-induced liver injury	Manufacturing complexity	[98]
Liposomes	Silymarin; HSPC; cholesterol	Improve stability; target liver	Size~122 nm; dose-dependent effects	Large-scale production costly	[101]
	Silymarin; PC; DCP; SA	Anti-inflammatory effects	AUC ↑ 3×; reduced IL-6, MPO	Physical instability	[102]
	Silybin; UAS; SL;	Inhibited alcohol-induced liver damage;	Size~133 nm; stability ↑; release 58%/24 h; ATP↑; Bcl-2 ↓; Bax ↓; Caspase 3 ↓;	Complex production process	[125]
Nanoemulsions	Silymarin; PEG 400; GMO; Cremophor	Improved solubilization and absorption	Improved ECG, reduced COX-2, TNF-α	Surfactant toxicity	[104]
Microemulsions	Silymarin; Labrafil; Transcutol; Tween 80	Solubility enhancement; brain delivery	Size 61 nm; release ↑ 92%/12 h; neuroprotection ↑	High surfactant content	[105]
SMEDDS/SNEDDS	Silymarin; ethyl linoleate; Tween 80	Solubilization; lymphatic uptake	Bioavailability ↑ 1.88× (solution) and ↑ 48.8× (suspension)	Excipient toxicity; regulatory concerns	[106]
	Silymarin; Cremophor; ethyl linoleate	Rapid dissolution	98.7%/60 min; 1.36× faster than Legalon^®^	High surfactant load	[76]
	Silymarin; oleoyl macrogol glycerides	Solubility & absorption ↑	Dissolution ↑ 13×; bioavailability ↑ 7.6×	Lymphatic transport unpredictable	[74]
Lipid Nanoparticles (SLNs/NLCs)	Silymarin; oleic acid; lecithin	Solubility, stability, uptake ↑	Size 83–107 nm; BA ↑ 75% (NLC), 60% (SLN)	Solid lipid crystallinity → drug expulsion	[108]
	Silymarin; glycerol distearates	Enhanced bioavailability	BA ↑ 2.5–3.1×	Scale-up challenges	[75]
Solid Lipid Nanoparticles	Silybin; Tween 80; Precirol	Improved stability & solubilization	BA ↑ 5–7× vs. fast-release	Low drug loading capacity	[79]
NLCs (Specialized)	Silymarin; cetyl palmitate; Lauroglycol	Permeability ↑	Papp ↑ 10×; 90 × 10^−6^ cm/s	High lipid content concerns	[78]
	Silymarin; Precirol; Labrafac	Targeting CNS	Brain deposition ↑ 12.46×	CNS targeting safety unknown	[114]
Polymeric Micelles	Silymarin; succinyl chitosan	Solubility ↑; controlled release	Release ↑ 81–87%; cell viability ↑ 2.8–3.3×	Polymer toxicity concerns	[117]
	Silymarin; Solutol HS15; Poloxamer F68	Solubility ↑	Solubility ↑ 460×; IC50 ↓	Surfactant crystallization risk	[118]
	Silybin; deoxycholate–GA micelles	Solubility ↑; liver targeting	Solubility ↑ 41×; AUC ↑ 4×	Synthesis complexity	[119]
	Silymarin; Pluronics	Sustained release	Release ↑ 95%/24 h; IC50 ↓ 5×	Rapid clearance	[121]

Note: Phosphatidylcholine (PC); hydrogenated soy phosphatidylcholine (HSPC); dicetyl phosphate (DCP); stearyl amine (SA); glyceryl monooleate (GMO); electrocardiogram (ECG); Cyclooxygenase-2 (COX-2); tumor necrosis factor (TNF-α); the central nervous system (CNS); glycyrrhetinic acid (GA); ursodeoxycholic acid sodium (UAS); soybean lecithin (SL); lactobionic acid (LA); N-(3-dimethylaminopropyl)-N’-ethylcarbodiimide hydrochloride (EDC); N-Hydroxysulfosuccinimide (Sulfo-NHS); cholesterol (Chol). ↑ means increased, ↓ means decreased, and → means leaded to or pointed to.

## Data Availability

Not applicable.
